# Genetic implications of Th/U, Th/K, and U/K ratios for U mineralizations: A case study from El-Missikat and El-Erediya shear zones, Eastern Desert, Egypt

**DOI:** 10.1186/s12932-023-00083-3

**Published:** 2023-10-17

**Authors:** Mahmoud Abdel-Hakeem, Mohamed El-Tahir, Ehab Abu Zeid, Hassan Rageh

**Affiliations:** 1 Department of Geology, Faculty of Science, South Valley University, Qena, Egypt; 2 Nuclear Materials Authority, Maadi, Cairo, Egypt

**Keywords:** El-Missikat mine, El-Erediya mine, U mineralization, Th/U ratio, Th/K ratio, U/K ratio

## Abstract

The current work is an attempt to reveal the possible utilization of the radiometric measurements to build-up a complete genetic scenario for magmatic, hydrothermal, and supergene uranium mineralization. For this purpose, ground gamma-ray survey was performed through the exploratory tunnels dug perpendicular to El-Missikat and El-Erediya shear zones, the Central Eastern Desert of Egypt. Contents of U, Th, and K were measured for the host pink granite (e.g., avg.15.94 U ppm, 35.62 Th ppm, and 6.63% K), alteration zones (brecciation, silicification, greisenization, kaolinization and hematitization) (e.g., avg. 124.01 U ppm, 63.67 Th ppm, and 3.13% K), and mineralized silica veins (e.g., avg. 312.65 U ppm, 92.22 Th ppm, and 2.62% K). All of these data were graphically represented as correlation plots of Th vs. U, Th/U vs. U, Th vs. K, and U/K vs. Th/K. The overall results indicate magmatic, hydrothermal, and supergene sources of El-Missikat and El-Erediya U mineralization. The magma-derived U contents are enclosed mainly in the pink granite that is mostly characterized by normal Th/U (2.5–5) and Th/K ratios (3–5*10^–4^). The hydrothermal processes through the alteration zones and mineralized silica veins are reflected by the weak correlation of Th with U (e.g. r = 0.13 and − 0.39), the strong negative correlation of Th/U ratio with U (e.g. r = − 0.82), 2.5˃Th/U˃0.1, Th/K˃5*10^–4^, Th/K < 3*10^–4^, and the strong positive correlation of U/K with Th/K (e.g. r = 0.91) as well as the occurrence of thorite, columbite, xenotime and hydrothermal zircon (0.5 > Th/U ≤ 0.1). Afterwards, the hydrothermal mineralization underwent some degrees of chemical weathering that resulted in supergene U mineralization whose fingerprints can be traced by the occurrence of secondary U minerals (e.g. kasolite and uranophane), Th/U ratios ≤ 0.1, and the weak correlation between Th/K and U/K (e.g. r = 0.39 and − 0.11).

## Introduction

U, Th, and K are considered to be large ion lithophile elements well-known for their radioactivity and heat production as well as the common occurrence, at relative abundances, in granites compared to the other rock types [8, 62]. They have a characteristic geochemical behavior (e.g. the incompatible magmatic fractionation of U and Th in relative to K and the post-magmatic mobilization of U and K compared to Th) under magmatic, hydrothermal, and supergene conditions (e.g [16, 18, 23, 24, 37, 47, 55, 59, 60], making them significant tracers in the geochemical exploration and mapping the hydrothermal and supergene alteration zones (e.g; [1, 13, 20, 26, 27, 36, 46, 48, 49, 51, 58, 64, 66, 67]. Apart from the other geochemical approaches such as fluid inclusions and whole rock geochemistry, the current work tries to build-up a radioelement ratio-based genetic scenario for vein-type uranium mineralizations by measuring Th/U, Th/K, and U/K ratios through host rocks, alteration zones, and U mineralized-veins. For this purpose, polymetallic, vein-type U mineralizations hosted by El-Missikat and El-Erediya pink granites, Eastern Desert of Egypt, were undertaken as a case study. The radioactivity of such localities was first discovered, under supervision of the Egyptian Authority of Nuclear Materials, using airborne gamma-ray survey during a project of uranium exploration between latitudes 25–27ºN, through the Central Eastern Desert of Egypt [7]. This was followed by ground inspection of these anomalous sites, leading to dig a number of exploratory mining tunnels through the northwestern part of El-Missikat pluton and the southern part of El-Erediya pluton [3, 4, 10, 31, 35]).

## Geological setting

**Fig. 1:**
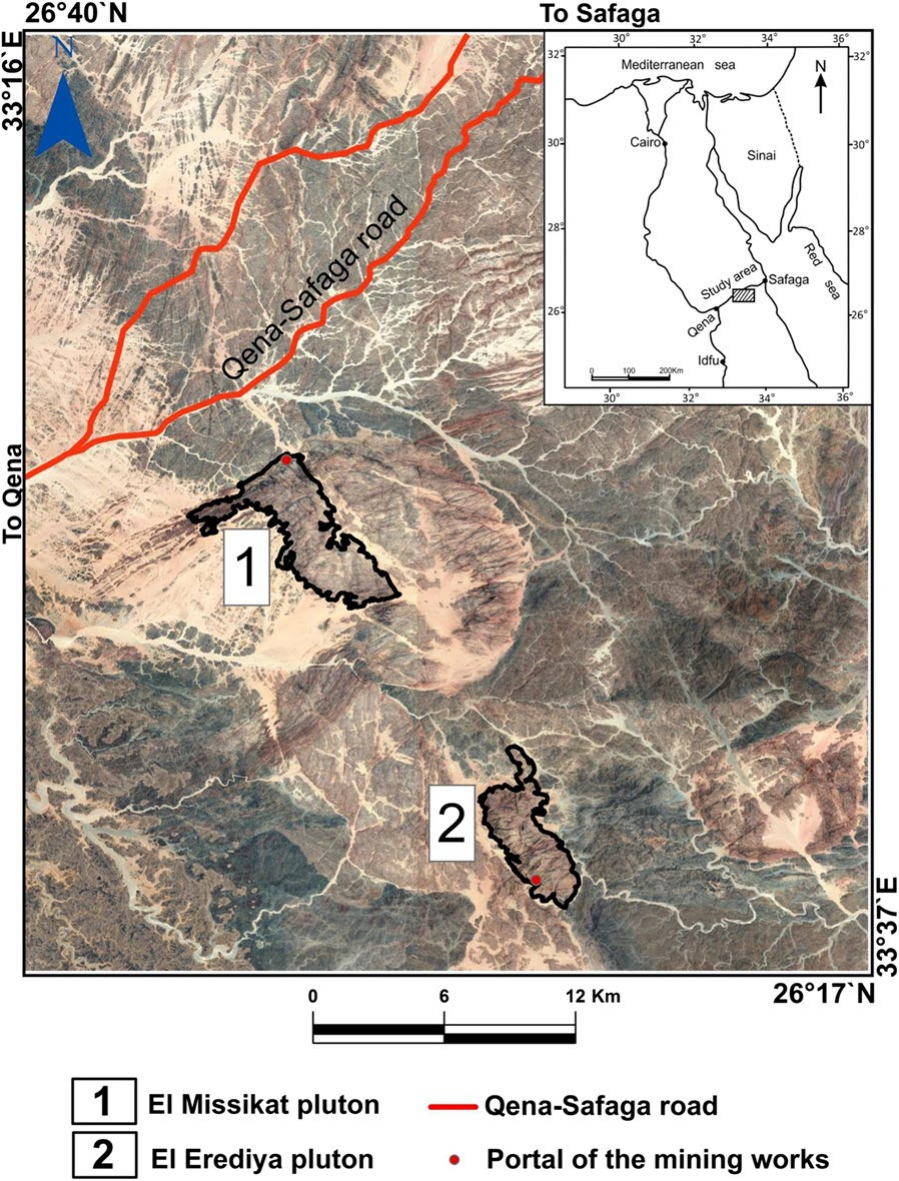
Location map of El-Missikat and El-Erediya plutons showing the portal of the mining works

**Fig. 2:**
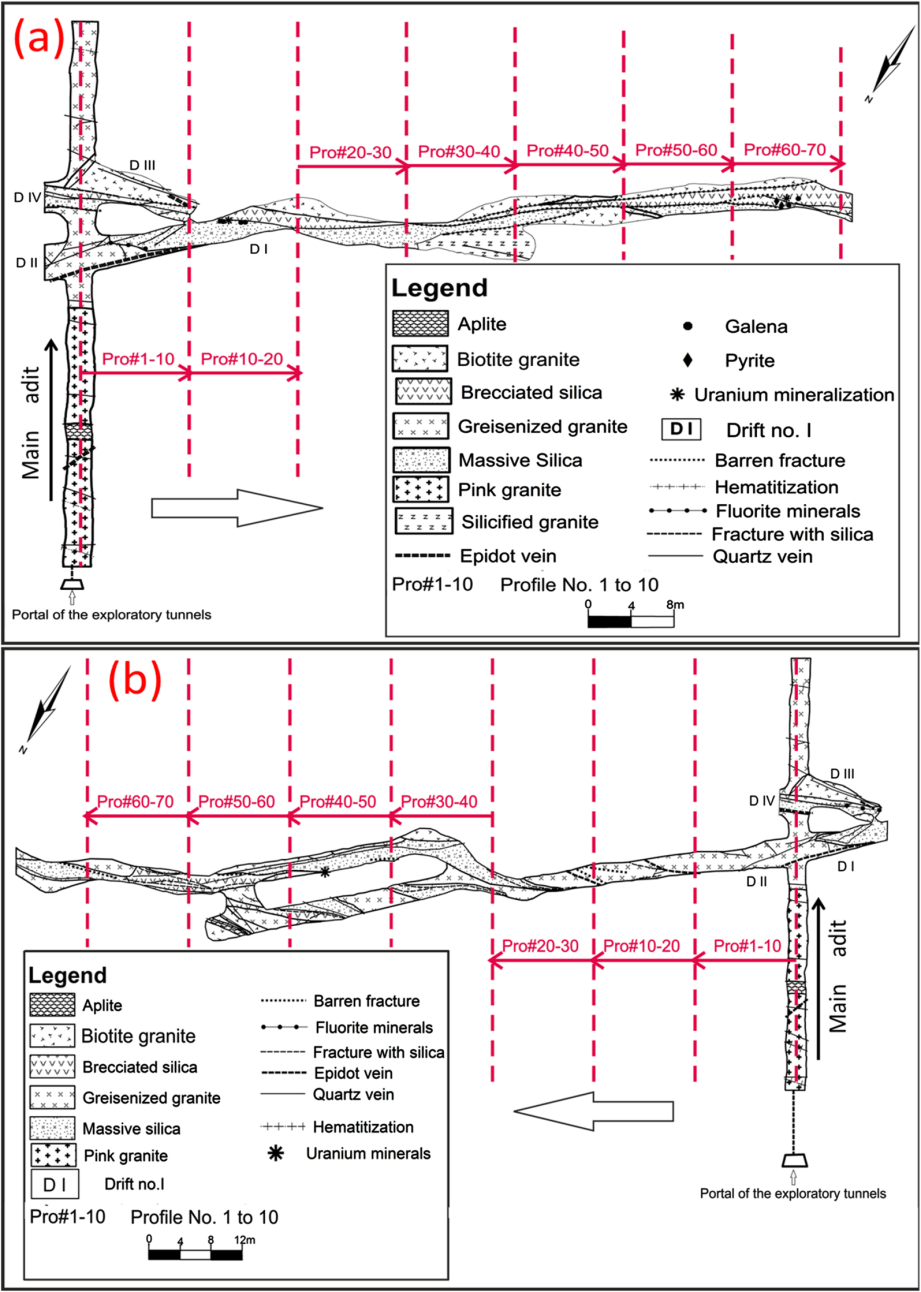
Geological maps of drifts# DI&DIII **a** and DII **b** through the northwestern part of El-Missikat pluton (after Abu Dief, 1985), with locations of the studied profiles (red dashed lines)

**Fig. 3:**
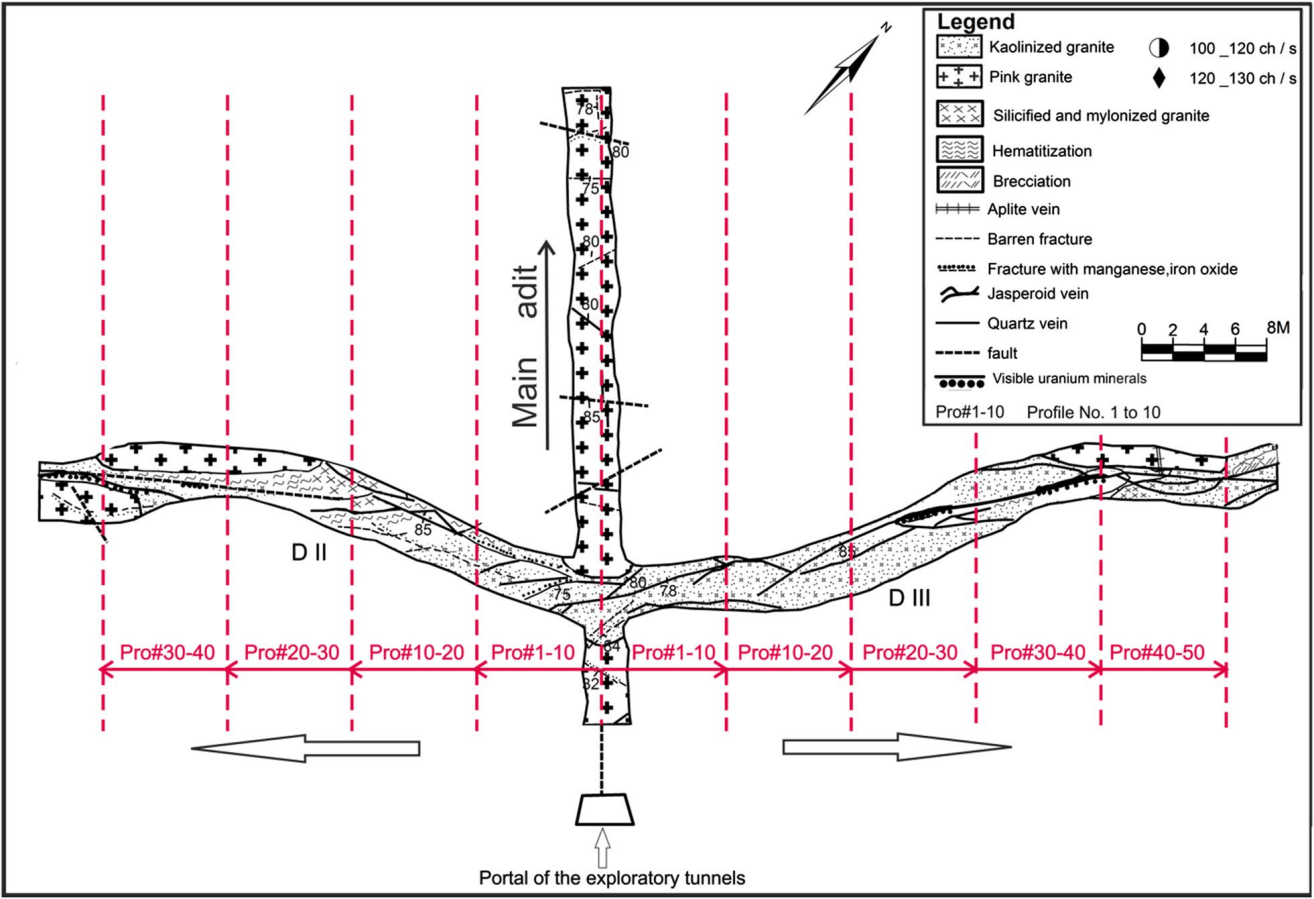
Geological maps of drifts# DII&DIII through the southern part of El-Erediya pluton (after El-Tahir, 1985), with locations of the studied profiles (red dashed lines)

**Fig. 4:**
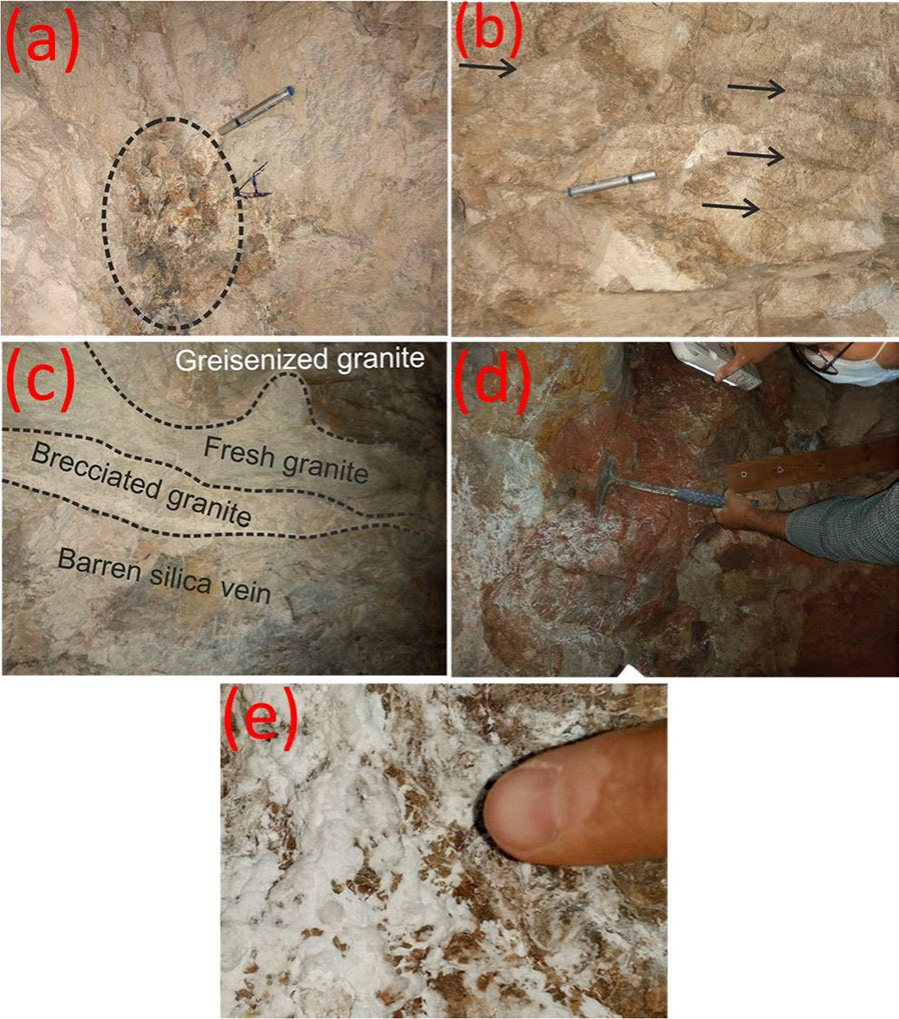
Field photographs show the alteration features of El-Missikat pink granite through DI and DII: **a** brecciation, **b** silicification, **c** greisenization, **d** hematitization, and **e** kaolinization

**Fig. 5:**
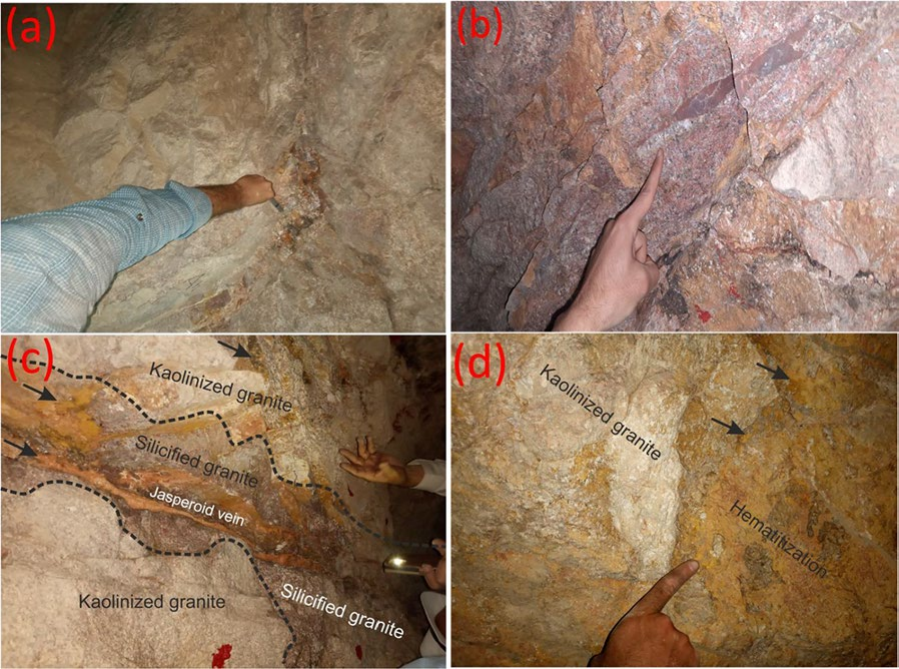
Field photographs show the alteration features of El-Erediya pink granite through DII and DIII: **a** brecciation, **b** silicification, **c** jasperiod vein in silicified granite, and **d** kaolinization stained by hematitization

**Fig. 6:**
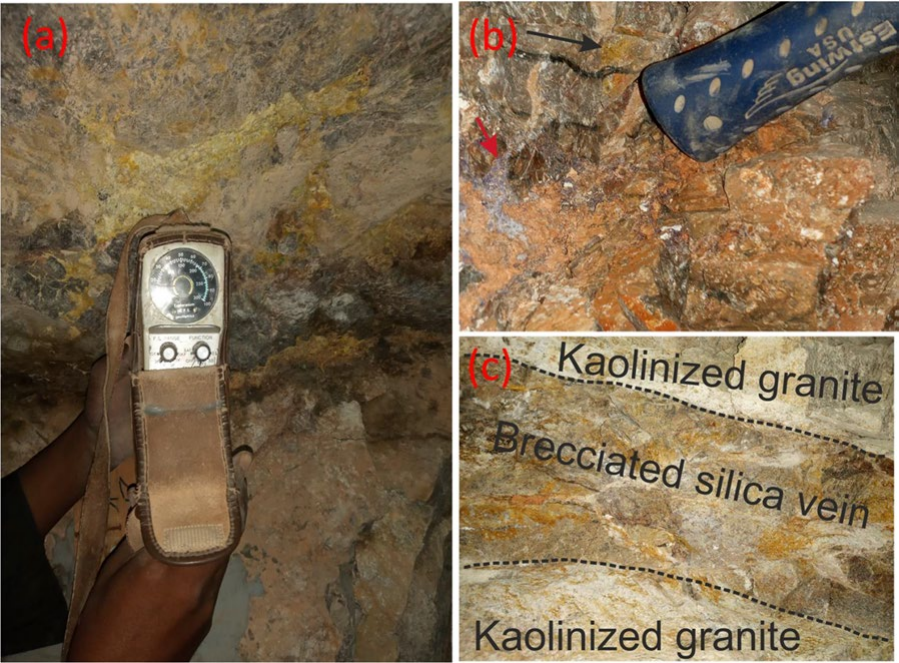
Field photographs show the occurrence of yellowish-greenish yellow secondary U minerals, at DI of El-Missikat shear zone, as fracture infill hosted by the brecciated silica veins **a** and associated with fluorite (**b**-look at red arrow), with occasional occurrence through the kaolinized granite (**c**)

**Fig. 7:**
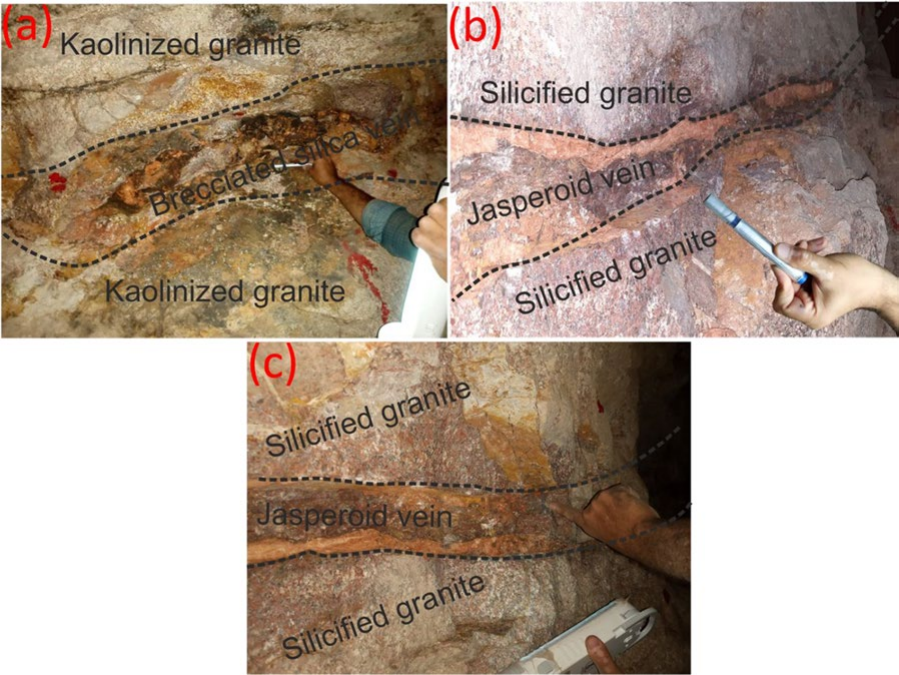
Field photographs show mineralized silica veins stained by yellowish secondary uranium minerals at DII of El-Erediya shear zone: **a** brecciated silica vein and **b** and **c** jasperoid veins

El-Missikat (26º 28ˋ 33ˋˋand 26º 29ˋ 50ˋˋ N and 33º 22ˋ 6ˋˋ and 33º 23ˋ 6ˋˋE) and El-Erediya pink granites (26º 18ˋ 35ˋˋand 26º 20ˋ 2ˋˋ N & longitudes 33º 28ˋ 10ˋˋ and 33º 29ˋ 43ˋˋE) are exposed as oval-shaped plutons elongated in NNW and NW directions, respectively, through the Central Eastern Desert of Egypt (Fig. 1). These plutons along with the hosted U mineralizations attracted several discussions [3, 4, 4, 5, 9–12, 30–35, 39, 41–43, 53, 56, 57, 63]. Accordingly, both El-Missikat and El-Erediya plutons are post-orogenic, peraluminous, medium-to-coarse-grained, younger granitic intrusions composed mainly of perthite (31–50%), plagioclase feldspars (15–20%), and smoky quartz (20–35%), with small amounts of biotite and hornblende (1–3%). Structurally, ENE-WSW and NE-SW trending shear zones cross cut El-Missikat and El-Erediya pink granites, respectively. Moreover, the exploratory mining tunnels through the northwestern part of El-Missikat (Fig. 2) and the southern part of El-Erediya plutons (Fig. 3) revealed that the pink granite is intruded by 1.5 m thick aplite dike at the main adit and suffers brecciation, silicification, greisenization, hematitization, and kaolinization (Figs. 4, 5). Toward the center of shear zone, parallel sets of red to black, lenticular-shaped, siliceous veins invading the pink granite of El-Missikat and El-Erediya areas, were also exposed. They host well-developed fluorite along with U mineralizations, represented mainly by pitchblende, uranophane, autunite, and soddyite, with varied thickness from few centimeters up to 10 m. It is worth to mention that the occurrence of pitchblende is less abundant due to its highly labile behavior under chemical weathering. So, the secondary U minerals, mostly yellowish to greenish in color, are commonly perceived during the field observations (Figs. 6, 7).

## Methodology

The distribution of U (ppm), Th (ppm), and ^40^ K (%) was determined for fresh granite, alteration zones, and radioactive siliceous veins within the exploratory tunnels by handheld gamma-ray spectrometer (RS-230). This device is characterized by BGO ‘‘bismuth germinate oxide’’detector by which the radioelement concentrations are directly measured without the need for calibration resources. The background was measured on the surface for the surrounding fresh granite, out of the exploratory tunnels, at 7.4 ppm U, 26.1 ppm Th, and 6% K. The radioactive measurements were systematically taken through successive profiles, 50 cm/read along each profile, that are perpendicular to shear zone and arranged at 1 m long distance, starting from the main adit until reaching the subsequent drifts (D_I_ and D_II_ at El-Missikat and D_II_ and D_III_ at El-Erediya). The total count of these measurements for fresh granite, brecciation, greisenization, silicification, kaolinization, hematitization, brecciated and massive silica veins was estimated at 27, 114, 390, 20, 43, 22, 226, and 231 reads. The obtained data set was then undergone statistically processing to study the varied correlations between them and drive the radio-elemental ratios, including Th/U, U/K, and Th/K. On the other hand, the radioactive measurements were coupled by separation of radioactive minerals from some alteration zones and mineralized silica veins at El-Missikat and El-Erediya. For this purpose, representative samples were ground up to − 500 μm and then bromoform-based separation along with handpicking was conducted. The separated mineral fraction was investigated by SEM–EDX. Although the current approach depends mainly on the radiometric measurements, its validity can be substantiated by literature (e.g. Heikal et al. 2022) that compared the radio-elemental ratios of the monzo-syenogranites (the Central Eastern Desert of Egypt)’’ obtained by the ground-gamma ray spectrometer (e.g. avg. 2.53 Th/U ratio) in relative to the data obtained by ICP-MS (e.g. avg. 2.9 Th/U ratio).

## Results and discussion

### Distribution of radioelements through pink granites

As listed in Table 1, it is clear that the distribution of U, Th, and K through El-Missikat pink granite is greatly similar to El-Erediya counterpart. Comparing with the normal averages in granites (2–8 ppm U, 10–30 ppm Th, and 3.6–4.5% K) [44, 55, 60], El-Missikat (15.94 ppm U, 39.21 ppm Th, and 6.63% K) and El-Erediya pink granite (16.05 ppm U, 35.62 ppm Th, and 7.07% K) can be considered as anomalously radioactive rock suites. The radioactivity is attributed mainly to the anomalous contents of U and Th due to the fact that K has radioactivity (e.g. 12–14 Ur) significantly lower than average granite (20 Ur) (Darnley, 1982). Th/U ratio is estimated at ranges of 1.30–3.5 (avg., 2.59) and 1.01–4.76 (avg., 2.42) for El-Missikat and El-Erediya pink granite, respectively. These values fall through the normal crustal ratio (2.5–5) [6, 21, 22], with some degree of U enrichment indicated by Th/U ratio below 2.5. Th/K ratio generally averages between 5.92*10^–4^ at El-Missikat and 5.05*10^–4^ at El-Erediya, and hence it can correspond to the normal crustal value of the unaltered lithologies, estimated at 5–3*10^–4^ [38, 64]. However, Th enrichment can result in higher Th/K ratios than the normal range (e.g. 8.78 at El-Missikat and 6.37 at El-Erediya).

**Table 1:** Distribution of U, Th, and K in pink granite through the main adit at El-Missikat and El-Erediya exploratory tunnels

Lithology/Alterations	U (ppm)	Th (ppm)	K (%)	Th/U	(Th/K)*10^–4^	(U/K)*10^–4^
El-Missikat	Avg., 15.94	39.21	6.63	2.59	5.92	2.41
	Max., 35.0	59.7	7	3.3	8.78	5.16
	Min., 10.0	26	6	1.3	4.12	1.43
El-Erediya	Avg., 16.05	35.62	7.07	2.42	5.05	2.29
	Max., 39.1	42.7	8.5	4.76	6.37	5.75
	Min., 7.4	26.1	6	1.01	3.68	1.03

### Distribution of radioelements through alteration zones

Altered granites are characterized by higher radio-measurements of U, Th, and K than that recorded for pink granite (Tables 2, 3). U has a general trend to be decreased from the brecciated granite (avg., 41.53–43.32 ppm at El-Missikat and 116.54–121.4 ppm at El-Erediya), away from the center of shear zone, toward the kaolinized zone (avg., 29.73–29.84 ppm at El-Missikat and 35.79–49.54 ppm at El-Erediya). The latter is generally enriched in U compared to pink granite; however, the low surface area of kaolinite limits the number of adsorption sites for U^+6^ ions carried by the invading solutions [69]. This can interpret the lower U values of the kaolinized granite compared to the other alteration zones, including brecciation (avg., 41.53–43.32 ppm at El-Missikat and 116.54–121.4 ppm at El-Erediya), silicification (avg., 41.63 ppm at El-Missikat), greisenization (avg., 36.11–41.77 ppm at El-Missikat), and hematitization (avg., 110.84–124.01 ppm at El-Erediya). Also, the maximum U values are measured from greisenization at El-Missikat (71.9–95.6 ppm) and hematitization at El-Erediya (184.6–266.1 ppm), complying with the hypothesis that greisenized and hematitc alterations are among the favorable environments for U accommodation (e.g. [19, 25].

**Table 2:** Distribution of U, Th, and K for the different alteration zones through drift# D_I_ and D#_II_ at El-Missikat exploratory tunnels

Lithology/alterations	U (ppm)	Th (ppm)	K (%)	Th/U	(Th/K)*10^–4^	(U/K)*10^–4^
D_I_						
Brecciation	Avg., 43.32	58.93	4.57	1.4	15.19	11.17
	Max., 69.2	71.6	7.3	2.29	49	37
	Min., 29.2	48.2	1.2	0.76	7.65	4.29
Silicification	Avg., 41.63	63.67	3.12	1.57	23.1	15.61
	Max., 59.1	71.9	6.7	2	31.38	26.86
	Min., 33.8	56.4	1.9	0.95	10.57	5.28
Greisenization	Avg., 41.77	57.32	4.48	1.45	13.82	10.08
	Max., 71.9	72.5	6	2.46	38.33	23.33
	Min., 26.4	44.5	1.5	0.78	8.56	4.56
Kaolinization	Avg., 29.73	56.21	4.15	1.93	14.34	7.44
	Max., 37.2	63.5	5.7	2.79	19.9	10.78
	Min., 22.1	49.2	2.7	1.48	8.63	5.14
D_II_						
Brecciation	Avg., 41.53	61.72	6.12	1.5	10.15	6.86
	Max., 45.0	72	7.6	1.79	12.82	8.42
	Min., 36.20	45.6	5.3	1.01	7.35	5.04
Greisenization	Avg., 36.11	56.4	4.46	1.64	14.34	9.38
	Max., 95.6	70.2	6.9	3.03	89.86	65
	Min., 19.0	36.3	0.7	0.6	8.05	4.45
Kaolinization	Avg., 29.84	54.89	4.9	1.87	11.57	6.32
	Max., 37.2	63	6.1	2.72	17.11	10.69
	Min., 23.2	45.4	3.2	1.4	8.05	4.62

**Table 3:** Distribution of U, Th, and K for the different alteration zones through drift# D_II_ and D#_III_ at El-Erediya exploratory tunnels

Lithology/Alterations	U (ppm)	Th (ppm)	K (%)	Th/U	(Th/K)*10^–4^	(U/K)*10^–4^
D_II_						
Brecciation	Avg., 121.4	32	6.01	0.35	5.38	21.49
	Max., 161.3	32.8	7.3	0.65	6.39	31.63
	Min., 47.2	30.6	5.1	0.2	4.19	6.47
Hematitization	Avg., 110.84	33.24	6.91	0.37	4.85	16.14
	Max., 266.1	41.8	8.2	0.67	6.47	32.45
	Min., 49.7	26.6	5.9	0.13	3.8	6.37
Kaolinization	Avg., 35.79	35.69	6.78	1.12	5.83	5.71
	Max., 89.2	102.5	8.3	2.91	51.25	29.7
	Min., 12.9	26.5	2	0.34	3.61	1.7
D_III_						
Brecciation	Avg., 116.54	39.44	6.56	0.38	6.04	17.78
	Max., 184.6	58.7	7.2	0.56	8.27	26.75
	Min., 61.6	29.3	5.3	0.16	4.25	9.78
Hematitization	Avg., 124.01	35.46	6.29	0.3	5.76	20.04
	Max., 184.6	43.8	7.1	0.42	7.08	26.75
	Min., 66.0	23.2	4.8	0.16	3.27	9.3
Kaolinization	Avg., 49.54	42.17	4.67	0.9	12.39	13.48
	Max., 95.8	219.5	8.5	7.42	199.5	41.14
	Min., 19.3	16.3	0.8	0.38	3.88	2.74

Th is measured through the alteration zones of El-Missikat at averages of 58.93–61.72 ppm, 63.67 ppm, 56.4–57.32 ppm, and 54.89–56.21 ppm corresponding to brecciation, silicification, greisenization, and kaolinization, respectively. Comparing with pink granite (avg., 39.21 ppm), there is a noticeable Th enrichment illustrated by Th/U > 1. Depending on the low mobilization behavior of Th in both the hypogene and supergene fluids [54], successive pulses of mineralized solutions are expected. On the other hand, Th values through El-Erediya alteration zones generally exhibit slight depletion (e.g. avg., 33.24–35.46 ppm for hematitization) in relative to pink granite (avg., 35.62 ppm) and the higher U enrichment, resulting in Th/U < 1. For K, its average values remarkably decline (e.g. brecciation ‘‘4.56–6.12%’’, silicification ‘’3.12%, greisenization ‘‘4.46–4.48%’’, and kaolinization ‘‘4.15–4.90%’’) away from that measured for pink granite at El-Missikat (avg., 6.63%), indicating moderately altered K-feldspar was imposed. Except for kaolinization (avg., 4.56%) through D_III_ at El-Erediya, slight changes of K contents (e.g. avg., 6.01–6.78%) in relative to pink granite (avg., 7.07%) are perceived. All of these changes in K contents are reflected by fluctuations of Th/K ratio above and below the normal value 5*10^–4^.

### Distribution of radioelements through silica veins

Through El-Missikat shear zone (Table 4), U contents in brecciated silica veins (e.g. avg., 88–121 ppm and max., 487–1007.7 ppm) are remarkably higher than the massive veins (e.g. avg., 54.66–82.19 ppm and max., 224.2–676 ppm), indicating other pulses of U-bearing hydrothermal solutions emplaced due to the reactivation of shear zone that in turn caused brecciation of the early-formed silica veins. On the other side, the red silica veins at El-Erediya are characterized by the highest U anomalies (e.g. avg., 183.26–312.65 ppm and max., 536.4–2990.5 ppm), probably due to the co-occurrence of silica and iron oxyhydroxides. The former is considered as a favorable repository for U accommodation that is facilitated by the latter whose task is to adsorb and reduce the dissolved U^+6^ ions [50].

**Table 4:** Distribution of U, Th, and K through U-mineralized silica veins at El-Missikat and El-Erediya exploratory tunnels

Lithology/alterations	U (ppm)	Th (ppm)	K (%)	Th/U	(Th/K)*10^–4^	(U/K)*10^–4^
Drift# D_I_						
El-Missikat tunnels						
Massive silica veins	Avg., 82.19	56.12	3.04	1.02	23.5	37.6
	Max., 676	76.2	6.3	2.11	195.33	200
	Min., 26.1	42.2	0.3	0.11	10.33	6.07
Brecciated silica veins	Avg., 121.1	54.65	3.07	0.86	20.37	41.44
	Max., 1007.7	78.8	6.4	1.81	68.6	185
	Min., 29.2	35.3	1	0.08	9.38	5.54
Drift# D_II_						
Massive silica veins	Avg., 54.66	51.81	4.34	1.13	12.53	51.06
	Max., 224.2	67.2	6.7	2.21	25.64	521.71
	Min., 23.4	38	2.5	0.22	7.68	6.65
Brecciated silica veins	Avg., 88.16	53.74	2.62	0.82	27.65	13.62
	Max., 487.0	72.1	5.6	1.71	120.8	89.68
	Min., 66.0	23.2	4.8	0.16	3.27	5.13
El-Erediya tunnels						
Drift# D_II_						
Red silica veins	Avg., 183.26	37.78	6.43	0.33	6.68	29.7
	Max., 536.4	95.2	9.8	1.35	36.62	92.48
	Min., 49.2	24.7	2.6	0.07	4.12	7.45
Drift# D_III_						
Red silica veins	Avg., 312.65	92.22	9.86	0.63	12.76	30.67
	Max., 2990.5	685.8	84.4	2.24	56.04	159.81
	Min.,41.5	23.5	2.4	0.06	4.37	8.56

### Genetic implications using Th/U ratio

**Fig. 8:**
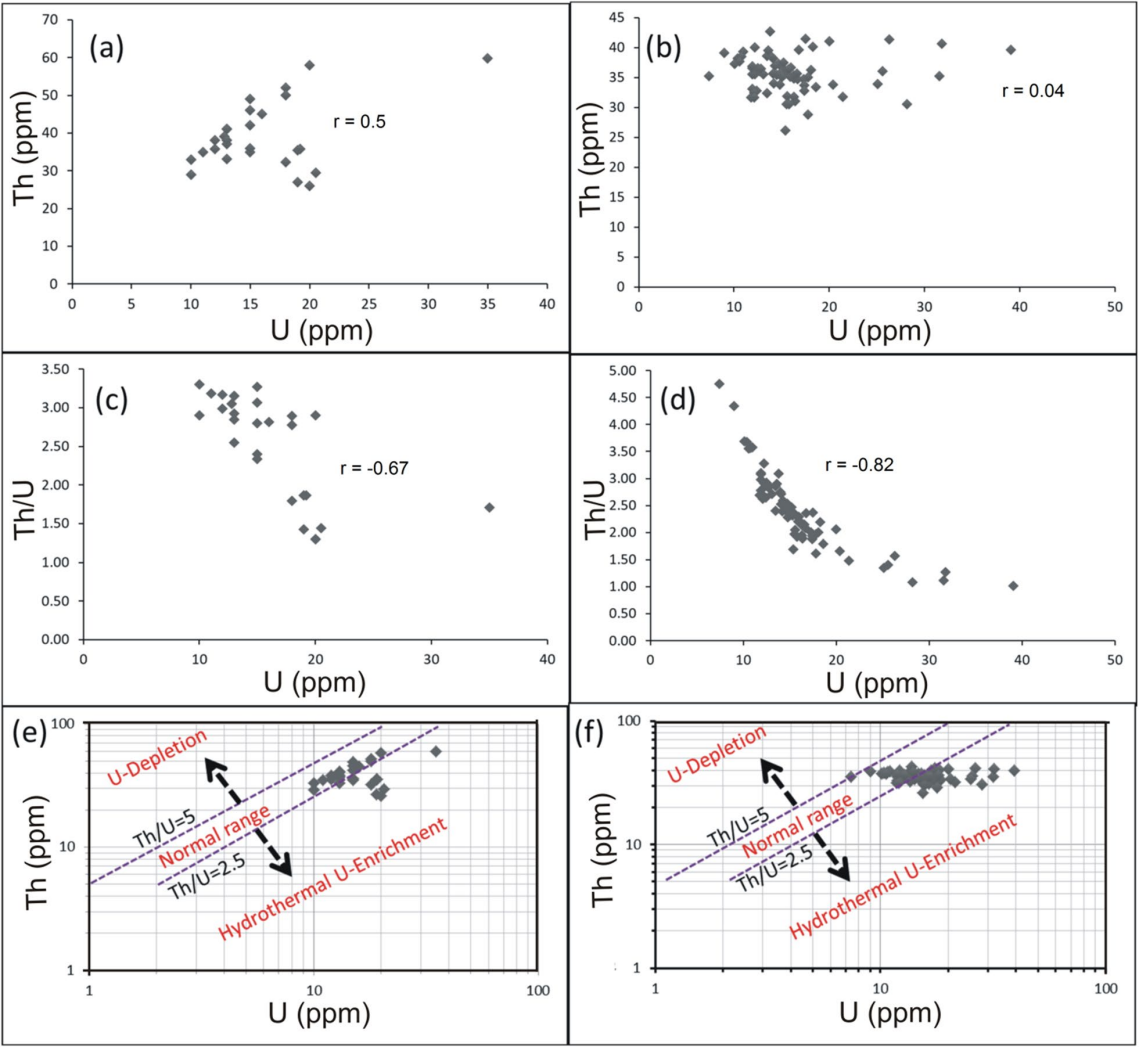
Binary plot of Th and Th/U ratio vs. U for El-Missikat **a** and **c** and El-Erediya pink granite **b** and **d**, along with variation diagrams (e- El-Missikat and f- El-Erediya) of Th/U ratio showing that the granite-contained U is of both magmatic (2.5–5 Th/U ration) and hydrothermal sources (Th/U<2.5)

U and Th are incompatible trace elements mostly concentrated in granitic magmas where they behave, without fractionation, as tetravalent ions related to each other. This relation is expressed as Th/U ratio that normally varies between 2.5 and 5 [6]. For the felsic magmatism, Th/U ratio is considered as a good indication for the magmatic differentiation degree between peralkaline and peraluminous granitic suites. In peralkaline magma the excess of alkalies in relative to alumina and the high temperature conditions induce high solubility of Th and U-bearing accessory minerals. Hence, the two elements are concentrated with each other and their Th/U ratio remains close to the average crustal ratio ~ 4. On the other side, the peraluminous conditions favor low solubility and fractionation of Th-bearing accessory minerals (e.g. monazite), resulting in an increase of U contents and Th/U ratios < 4 [23]. In all cases, Th and U contents of magmatic origin reveal a strong positive correlation. Comparing with the current study, pink granites of El-Missikat (Fig. 8a) and El-Erediya (Fig. 8b) exhibit moderate and very weak relationship between Th and U at correlation coefficients of 0.5 and 0.04, respectively, indicating U enrichment. Also, U contents exhibit a strong negative correlation with Th/U ratios for El-Missikat (r = − 0.67) (Fig. 8c) and El-Erediya pink granite (r = − 0.82) (Fig. 8d). Further, the calculated Th/U ratios fall through the normal range and below 2.5. Accordingly, magmatic and post-magmatic origin can be presumed for the granite-hosted U mineralization. The post-magmatic source can be either hydrothermal or supergene solutions. The former can be discriminated from the latter using Th/U ratio whose range ‘‘2.5 > Th/U > 0.1’’ is assigned to the hydrothermal uranium mineralizations [17, 40, 68], while values ≤ 0.1 are associated with supergene enrichment [15, 16]. These values, along with the normal ratio, are collectively represented by Th-U variation diagram on which both magmatic and hydrothermal sources are assigned to the anomalous radioactivity of the studied granite at El-Missikat (Fig. 8e) and El-Erediya (Fig. 8f). Another clue can be driven from Th/U ratios measured by Mohammed [52] for zircon crystals separated from El-Missikat ‘‘0.24’’ and El-Erediya granite ‘‘0.30’’. Discussion of these values here according to the recent works (e.g. [45, 61, 70, 71] that categorized zircon into magmatic ‘‘Th/U > 0.5’’, hydrothermal “0.5 > Th/U ≤ 0.1”, and metamorphic variety ‘‘Th/U ≤ 0.1’’, supports the aforementioned hydrothermal origin.

**Fig. 9:**
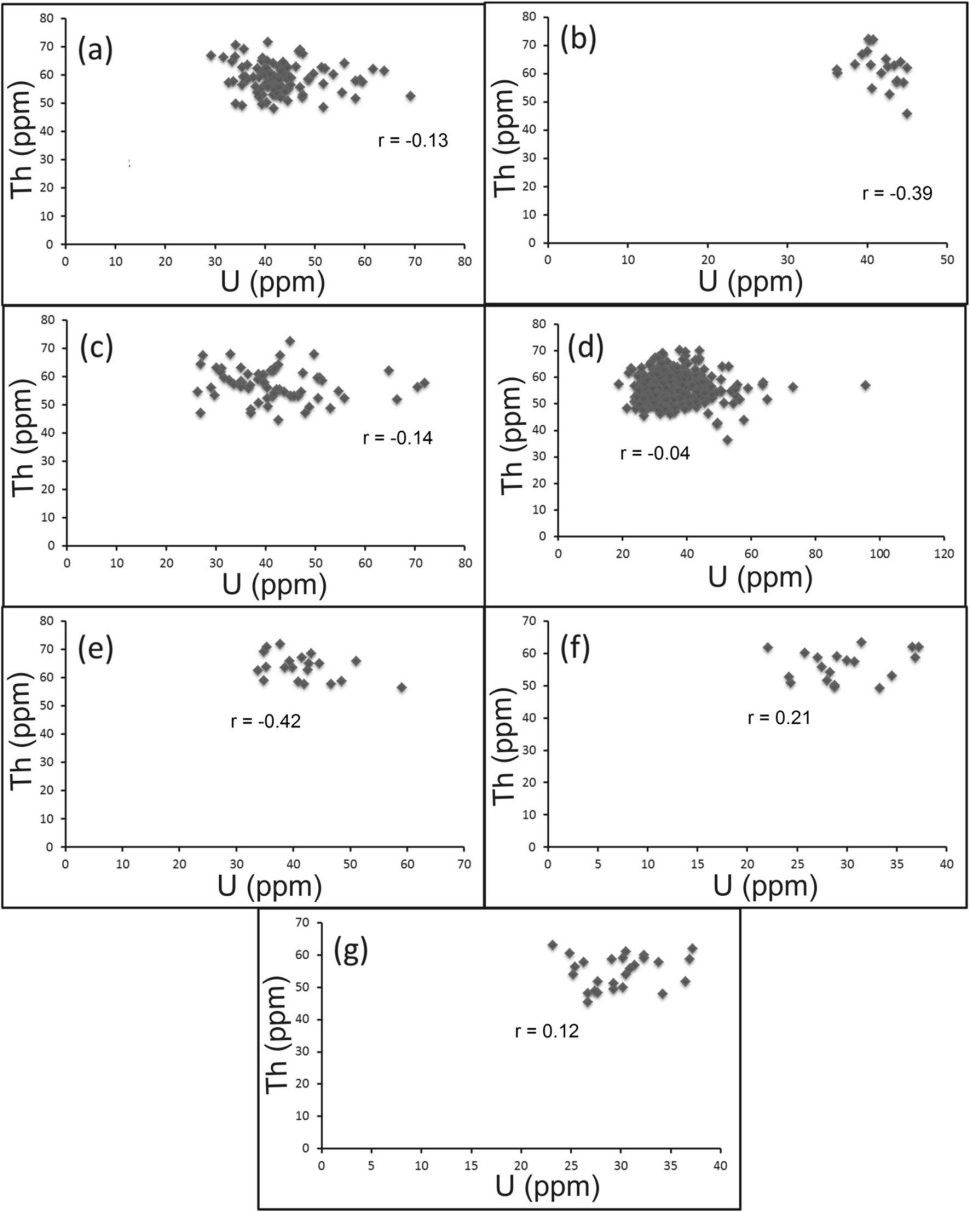
Binary plot of Th vs. U through the different alteration zones at El-Missikat drifts# DI **a**, **c**, **e**, and **f** assigned to brecciation, greisenization, silicification, and kaolinization) and DII (**b**, **d**, and **g** assigned to brecciation, greisenization, and kaolinization) showing weak correlation coefficients as an indication for U-enrichment

**Fig. 10:**
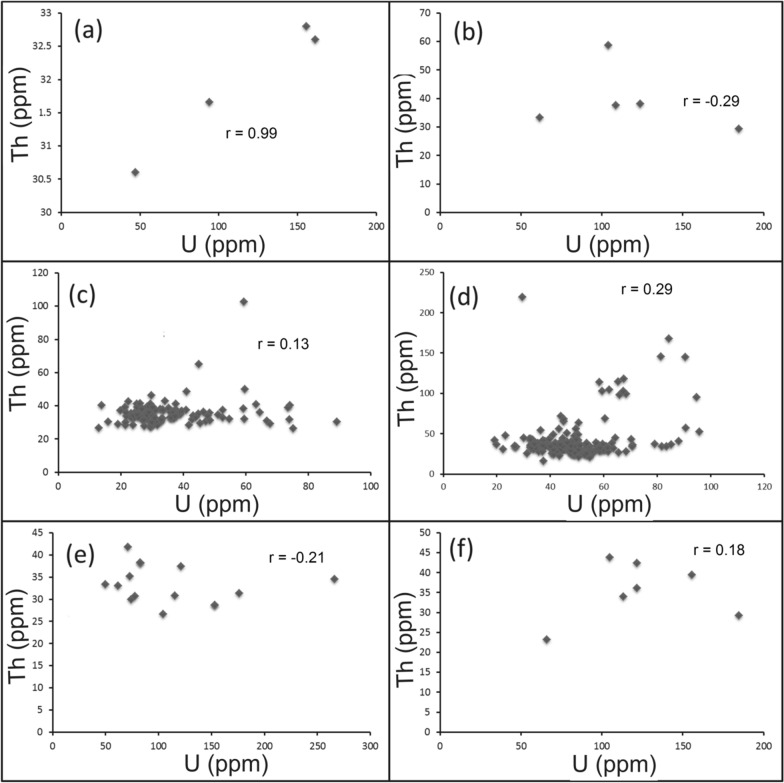
Binary plot of Th vs. U through the different alteration zones at El-Erediya drifts# DII **a**, **c**, and **e** assigned to brecciation, kaolinization, and hematitization) and DIII **b**, **d**, and **f** assigned to the same order) showing weak correlation coefficients as an indication for U-enrichment

**Fig. 11:**
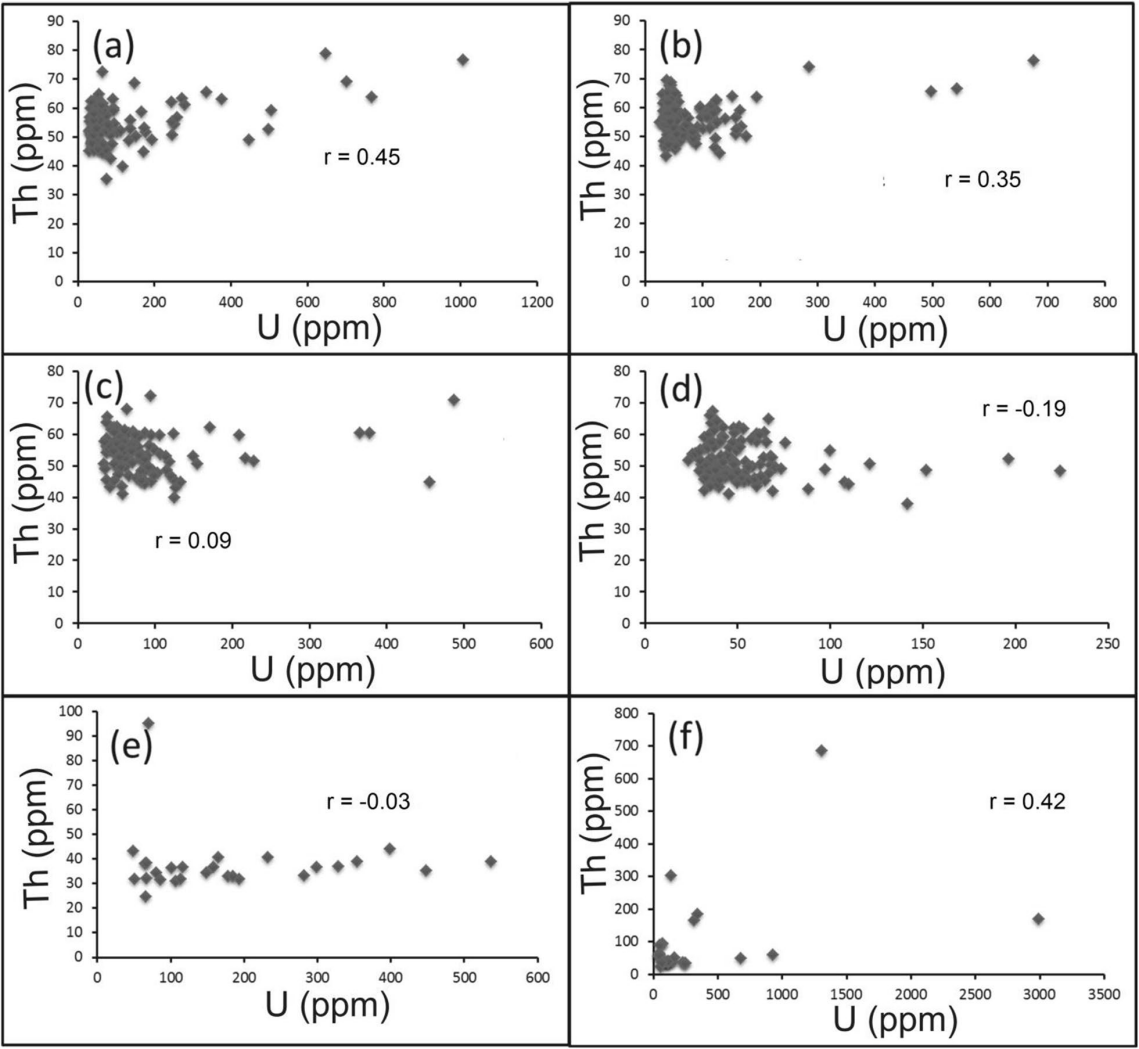
Binary plot of Th vs. U through varied siliceous veins at El-Missikat (**a** and **b** brecciated and massive silica veins through drift#DI and **c** and **d** brecciated and massive silica veins through drift#DII) and El-Erediya (e &f reddish silica veins through drfits# DII and DIII)

**Fig. 12:**
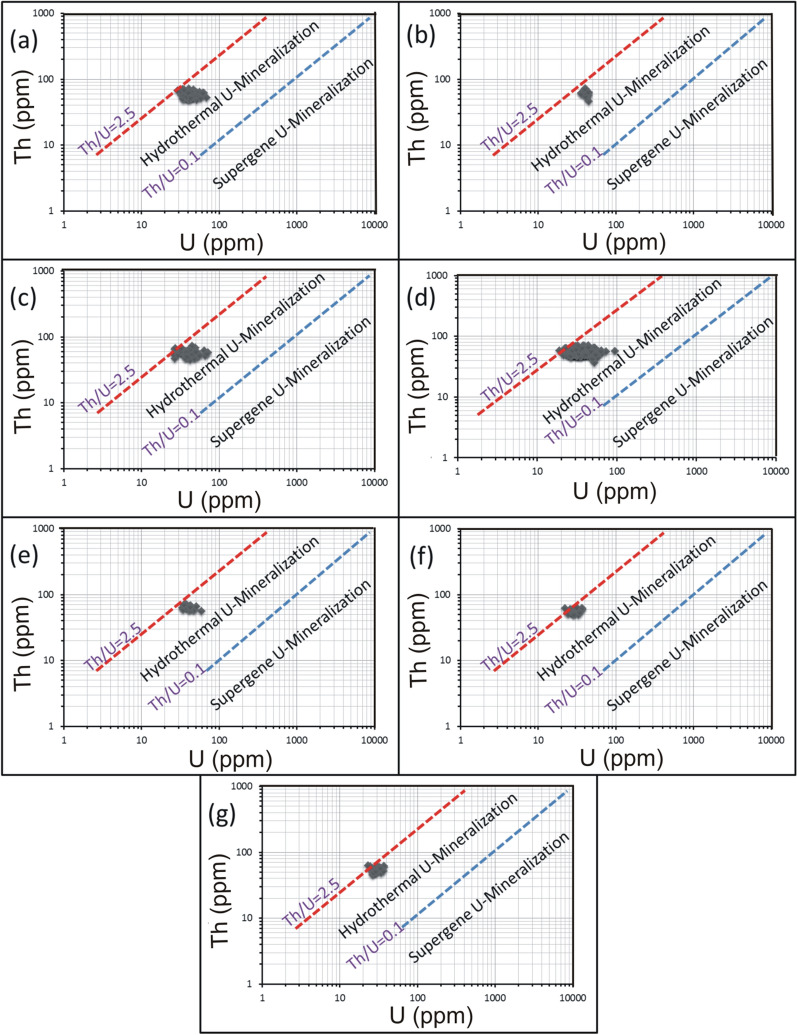
Th-U variation diagrams of El-Missikat alteration zones at drifts#DI (**a**, **c**, **e**, and **f** assigned to brecciation, greisenization, silicification, and kaolinization) and DII (**b**, **d**, and **g** assigned to brecciation, greisenization, and kaolinization) discriminate between hydrothermal (2.5 > Th/U > 0.1) and supergene U-mineralization (Th/U ratios ≤ 0.1)

**Fig. 13:**
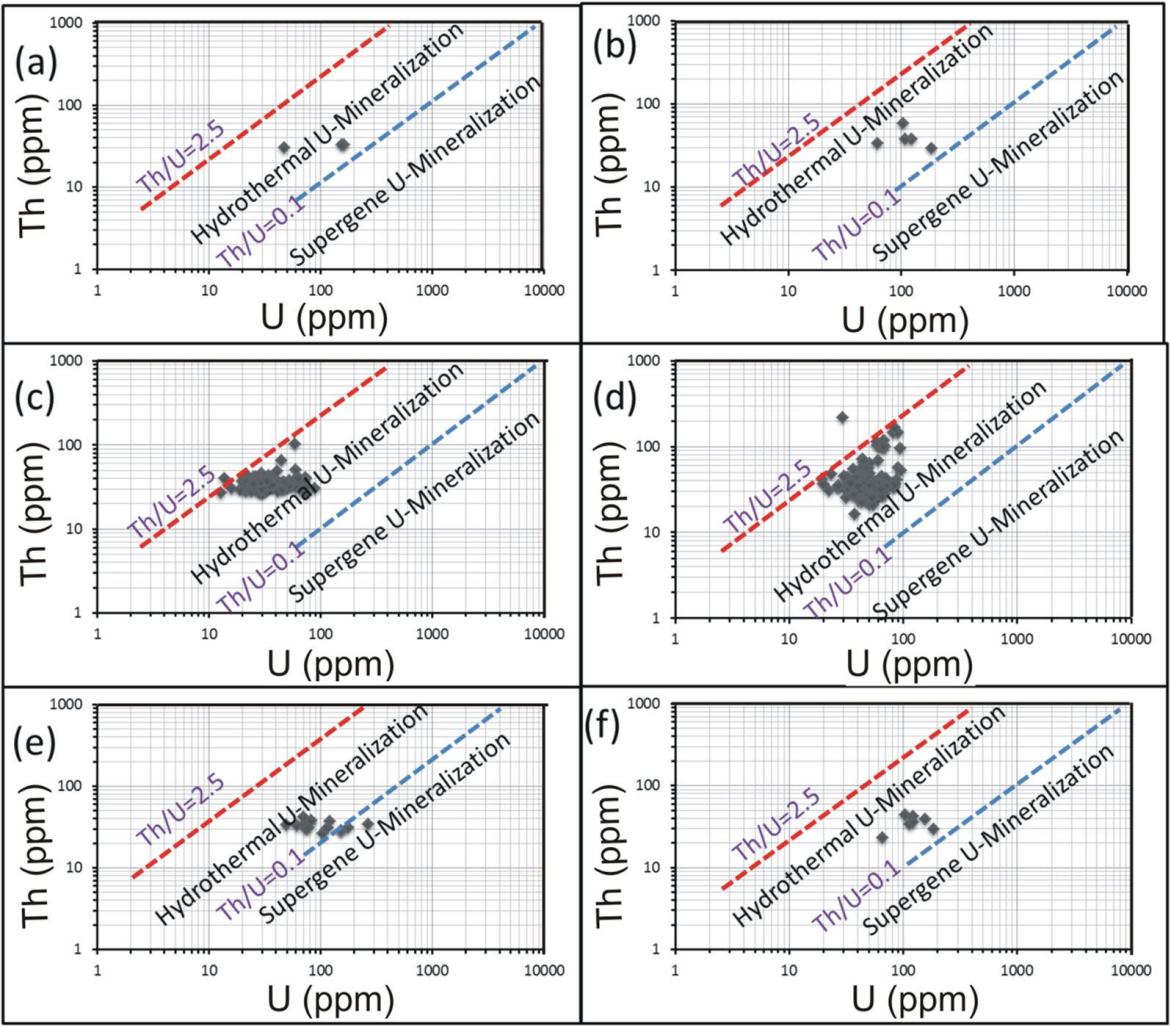
Th-U variation diagrams of El-Erediya alteration zones at drifts# DII (**a**, **c**, and **e** assigned to brecciation, kaolinization, and hematitization) and DIII (**b**, **d**, and f assigned to the same order) discriminate between hydrothermal (2.5 > Th/U > 0.1) and supergene U-mineralization (Th/U ratios ≤ 0.1)

**Fig. 14:**
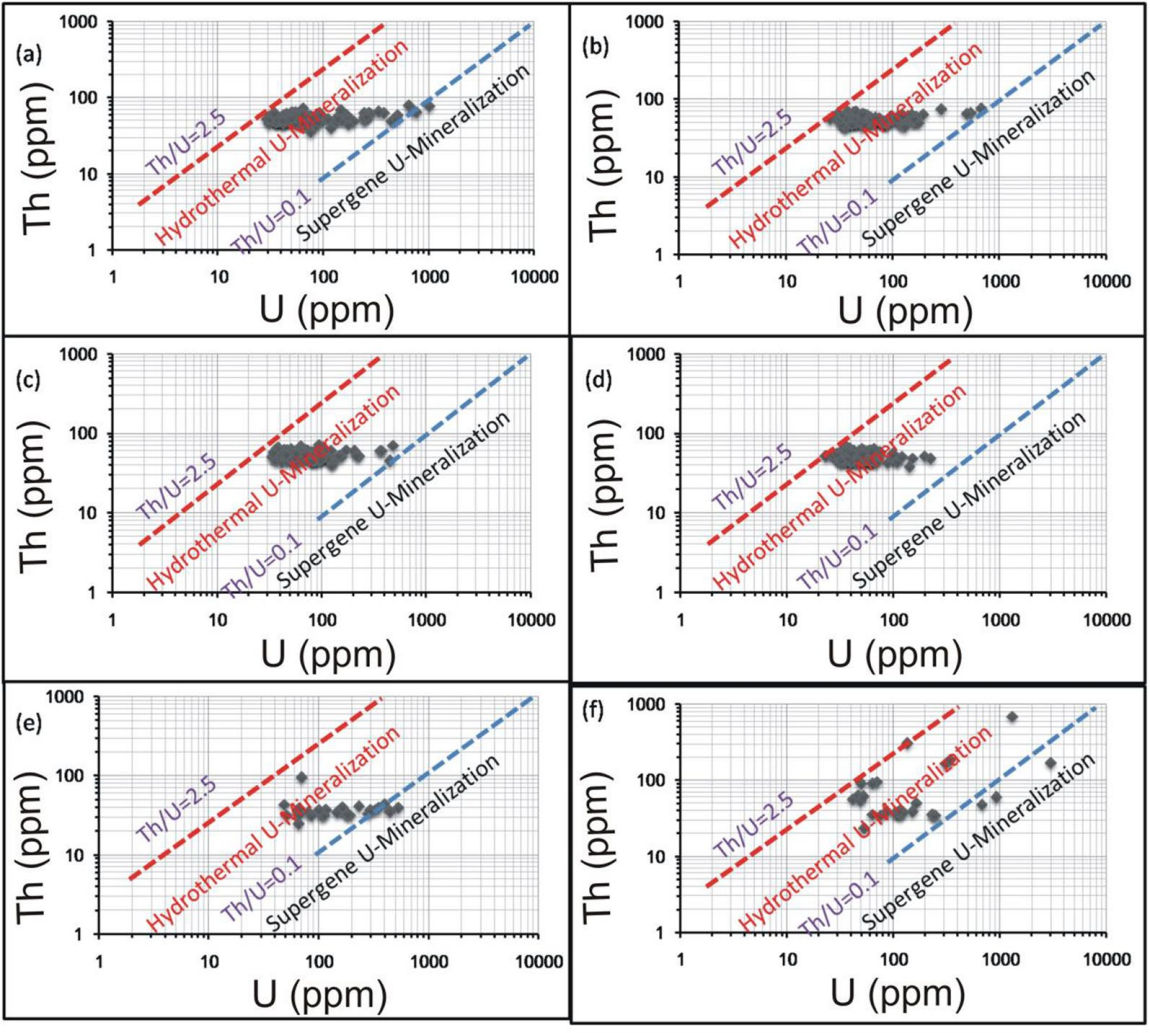
Th-U variation diagrams of the mineralized silica veins at El-Missikat (**a** and **b** brecciated and massive silica veins through drift#DI and **c** and **d** brecciated and massive silica veins through drift#DII) and El-Erediya (**e** and **f** reddish silica veins through drfits# DII and DIII) discriminate between hydrothermal (2.5 > Th/U > 0.1) and supergene U-mineralization (Th/U ratios ≤ 0.1)

For alteration zones and mineralized silica veins, U enrichment is also reflected by the weak correlation between Th and U (Figs. 9,10, 11). On Th-U variation diagram, most of the data are concentrated through the hydrothermal field, with an exception for the brecciated silica veins at El-Missikat as well as red silica veins and hematitization at El-Erediya where some samples fall through the field of supergene enrichment (Figs.12, 13, 14). Besides radiometric measurements, the mineral separation for the highly anomalous, mineralized-vein samples revealed the occurrence of some hydrothermally sourced, radioactive minerals, including thorite, betafite, coffinite, and zircon, and some supergene U minerals represented mainly by uranophane and kasolite (Table 5). The aforementioned hydrothermal minerals probably refer to the magmatic source of some pluses of the invading hydrothermal solutions. This claim is suggested according to a number of facts, including the concentration of Th in the residual magmatic fluids, the occurrence of Th-rich minerals (e.g. thorite, and zircon), and the poor liberation behavior of Th from its host rocks by hydrothermal solutions.

**Table 5:** EDX analysis of the separated radioactive minerals from some altered granites and silica veins at El-Missikat and El-Erediya exploratory tunnels

Elements %	Thorite		Betafite		Zircon		Coffinite		Uranophane		Kasolite
	A	B	C	D	E	F	G	H	I	J	K
SiO_2_	34.82	28.30	–	–	45.9	42.2	25.2	23.8	38.9	53.92	43.88
UO_2_	8.55	10.90	36.9	28.7	4.2	3.4	65.8	67.2	53.7	33.73	30.03
ThO_2_	42.29	49.12	1.22	2.4	1.07	1.3	–	–	–	–	–
Al2O_3_	2.77	1.97	5.77	3.45	7.30	6.2	0.65	0.12	3.67	5.08	4.8
TiO_2_	–	–	25.5	29.2	–	–	–	–	–	–	–
CaO	–	0.42	1.4	1.08	1.95	2.7	1.2	2.5	1.0	1.97	–
PbO	–	0.30	–	0.10	–	–	2.3	2.51	0.1	0.2	20.17
FeO	2.98	0.25	2.35	2.16	1.58	–	0.9	1.7	1.5	4.32	–
BaO	–	–	0.08	0.48	–	–	0.5	0.9	–	–	–
ZrO_2_	–	–	–	–	30.9	35.5	–	–	0.1	0.4	–
Nd2O_3_	–	–	2.5	3.7	–	–	0.14	0.11	–	–	–
Y2O_3_	7.59	8.23	0.89	0.45	5.78	6.0	1.05	0.83	–	–	–
Ce2O_3_	–	–	1.53	2.78	–	–	0.29	0.28	–	–	–
Ta2O_5_	–	–	3.41	4.51	–	–	–	–	–	–	–
Nb2O5	–	–	16.6	20.7	–	–	–	–	–	–	–
Total	99.0	99.49	98.2	99.7	98.6	97.3	98.03	99.9	98.97	99.62	98.88

^*^A—Greisenization through DII of El-Missikat; B—Kaolinization through DIII of El-Erediyal; C—red silica veins through DII of El-Erediya; D—brecciated silica veins through DI of El-Missikat; E—silicification through DI of El-Missikat; F—brecciated granite through DII of El-Erediya; G and H—massive and brecciated silica veins, respectively, through DII of El-Missikat; I and J—hematitization and red silica veins, respectively, through DIII of El-Erediya; K—brecciated silica veins through DII of El-Missikat

### Genetic implications using Th/K ratio

**Fig. 15:**
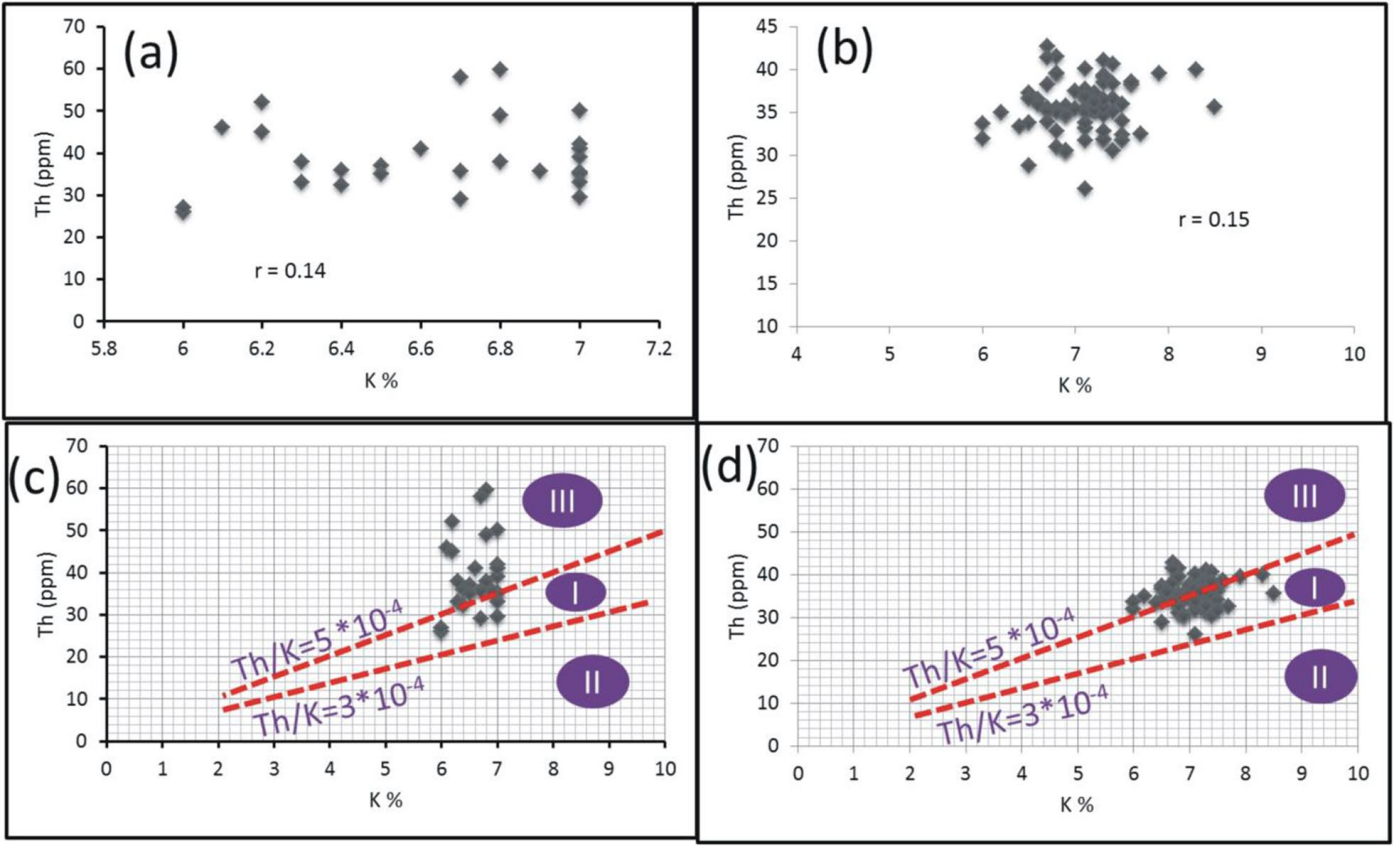
Th-K binary plot shows weak correlations of El-Missikat **a** and El-Erediya pink granite **b**, along with Th-K variation diagrams reveal normal Th/K ratio (I) (3–5*10–4) and Th-enrichment (III) (Th/K > 5*10–4) of El-Missikat **c** and El-Erediya pink granite **d**, without any signs for K-metasomatism (II) (Th/K< 3*10–4)

**Fig. 16:**
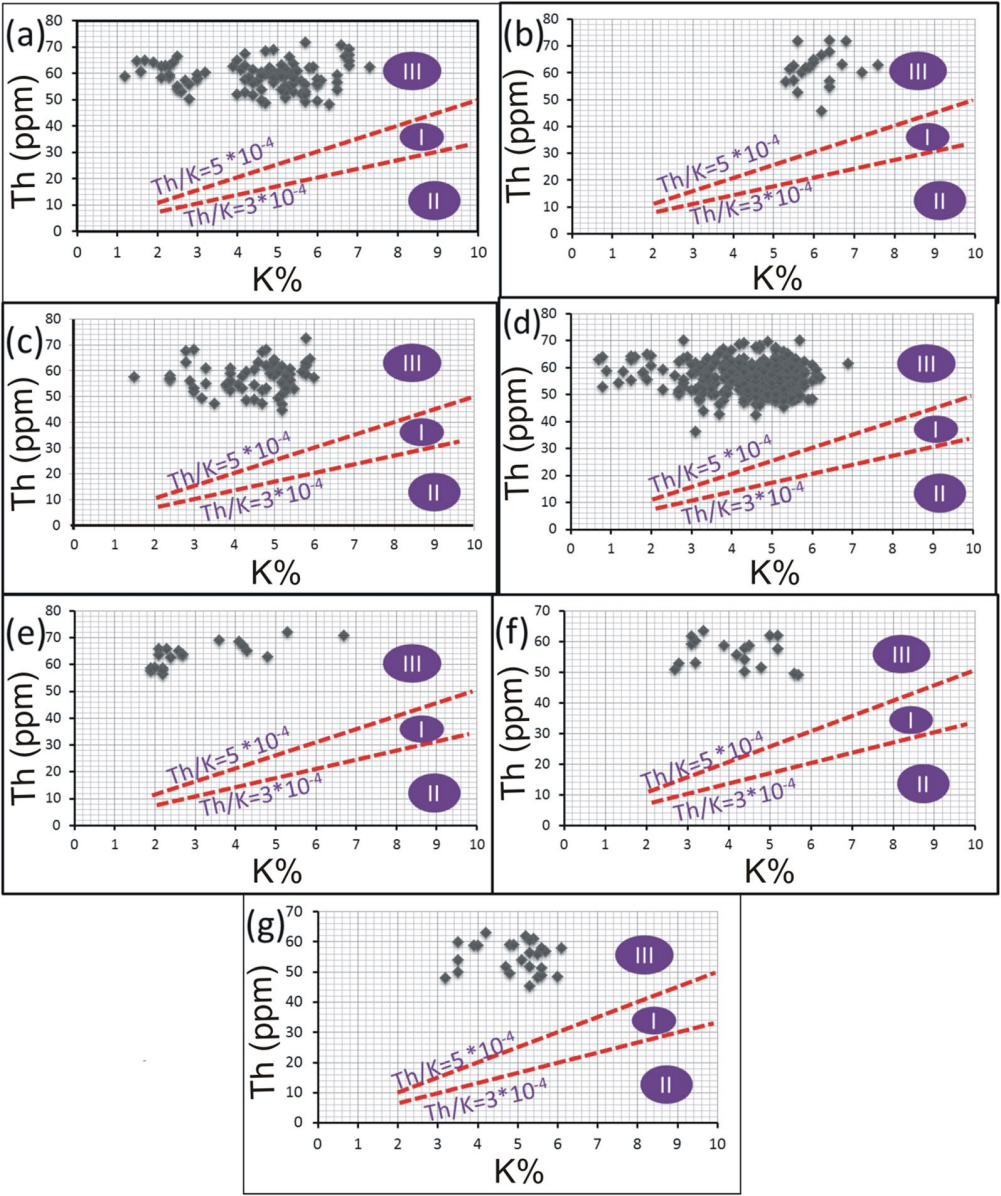
Th-K variation diagrams indicate the dominance of Th-enrichment (III) through the alteration zones of El-Missikat exploratory tunnels (**a** and **b** brecciation, **c** and **d** greisenization, **e** silicification, **f** and **g** kaolinization)

**Fig. 17:**
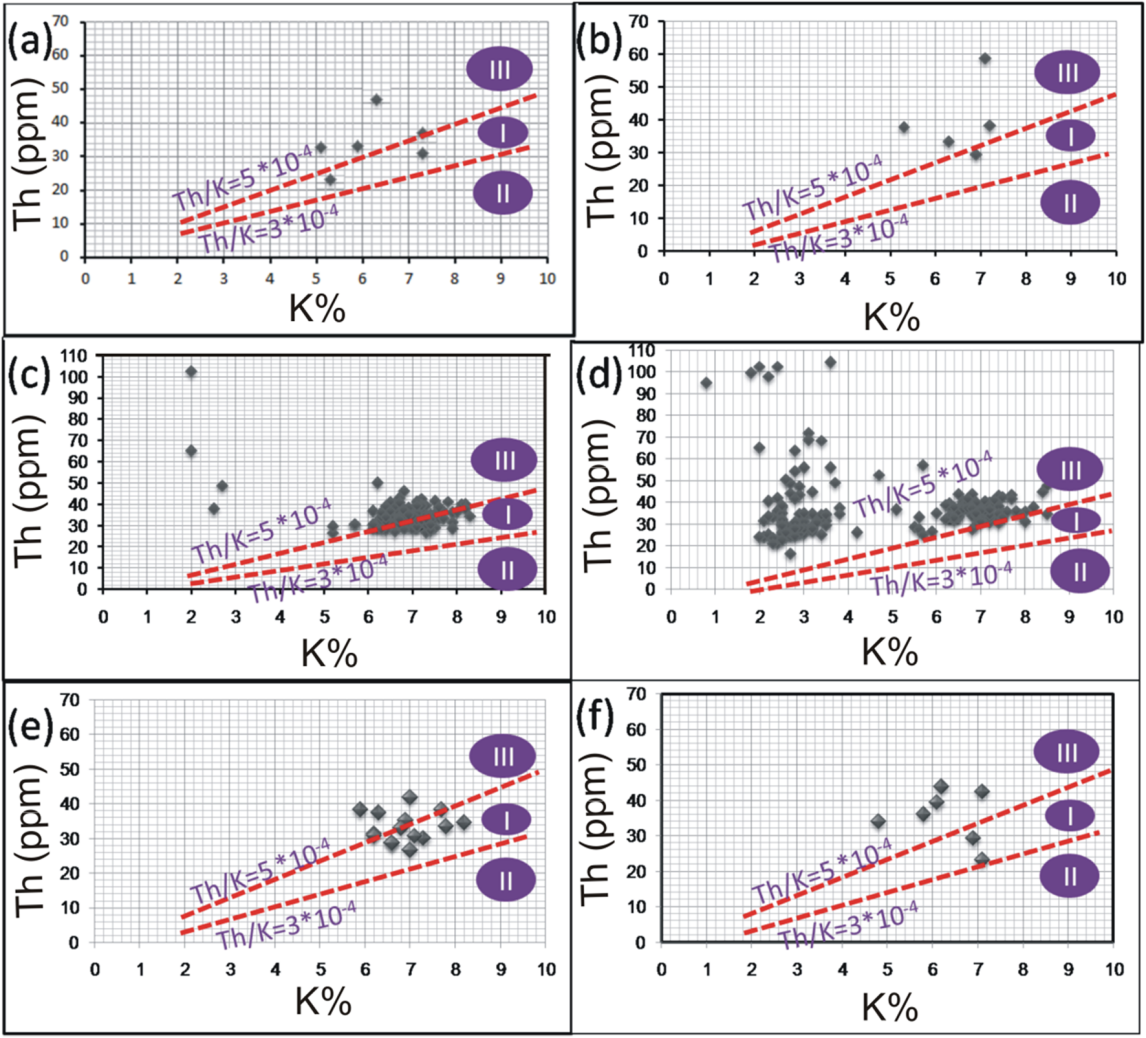
Th-K variation diagrams show that most measurements through El-Erediya alteration zone (**a** and **b** brecciation, **c** and **d** kaolinization, **e** and **f** hematitization) fall through the normal range (I) and Th-enrichment (III)

**Fig. 18:**
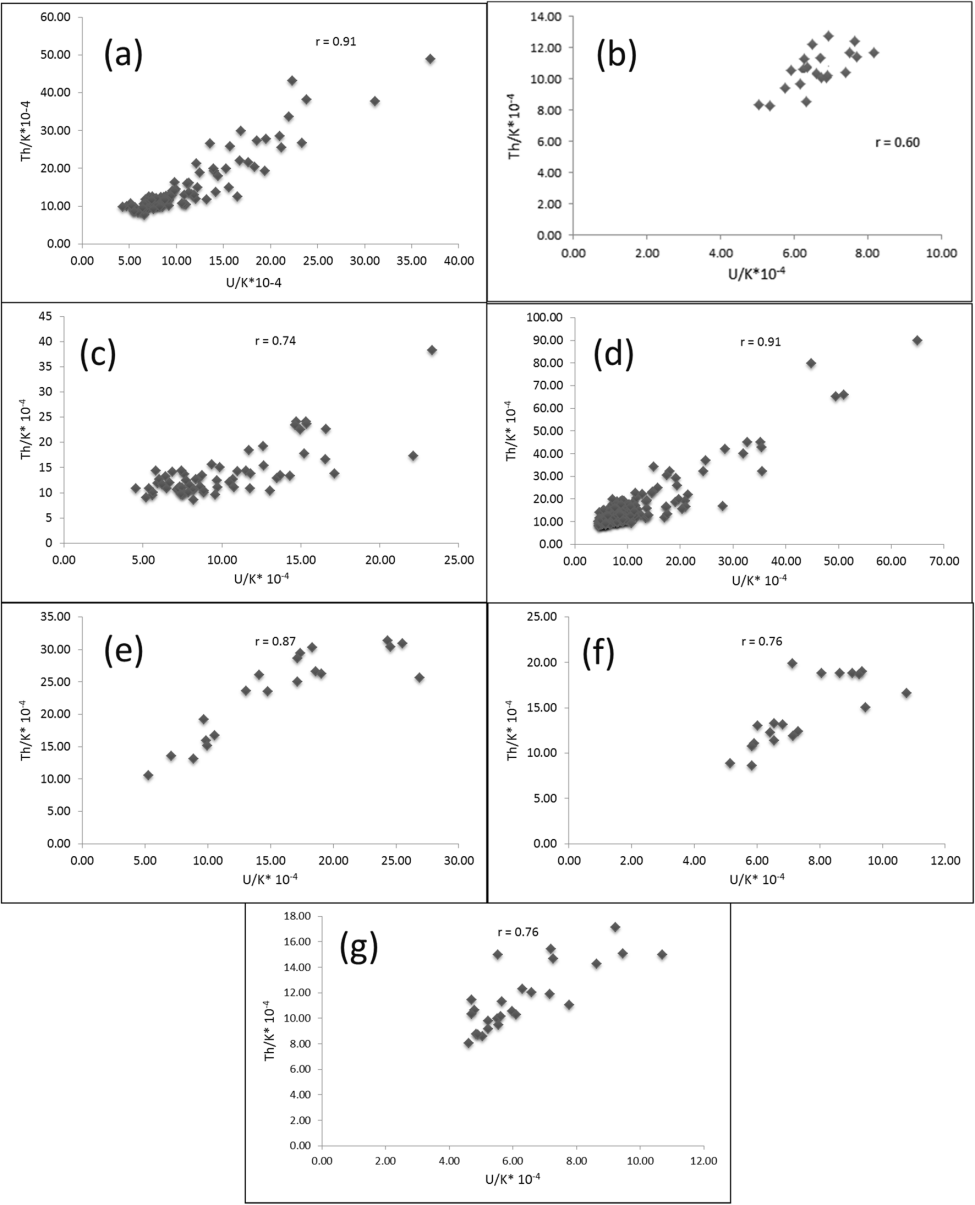
Binary plot of Th/K vs. U/K ratios showing strong positive correlations through the alteration zones of El-Missikat exploratory tunnels (**a** and **b**-brecciation, **c** and **d**-greisenization, **e**-silicification, and **f** and **g**-kaolinization)

**Fig. 19:**
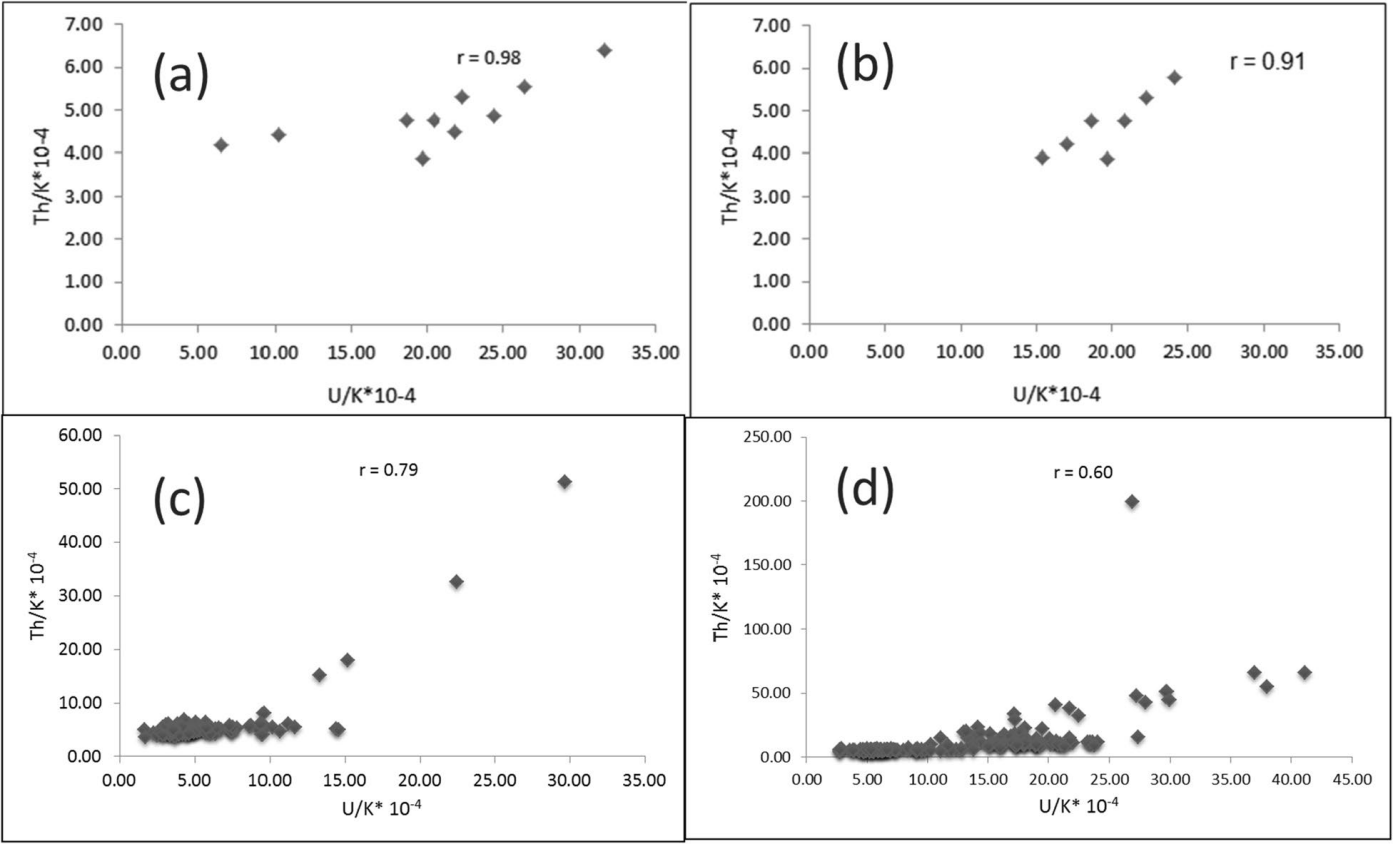
Binary plot of Th/K vs. U/K ratios showing strong positive correlations through the alteration zones of El-Erediya exploratory tunnels (**a** and **b**-brecciation and c&d-kaolinization)

**Fig. 20:**
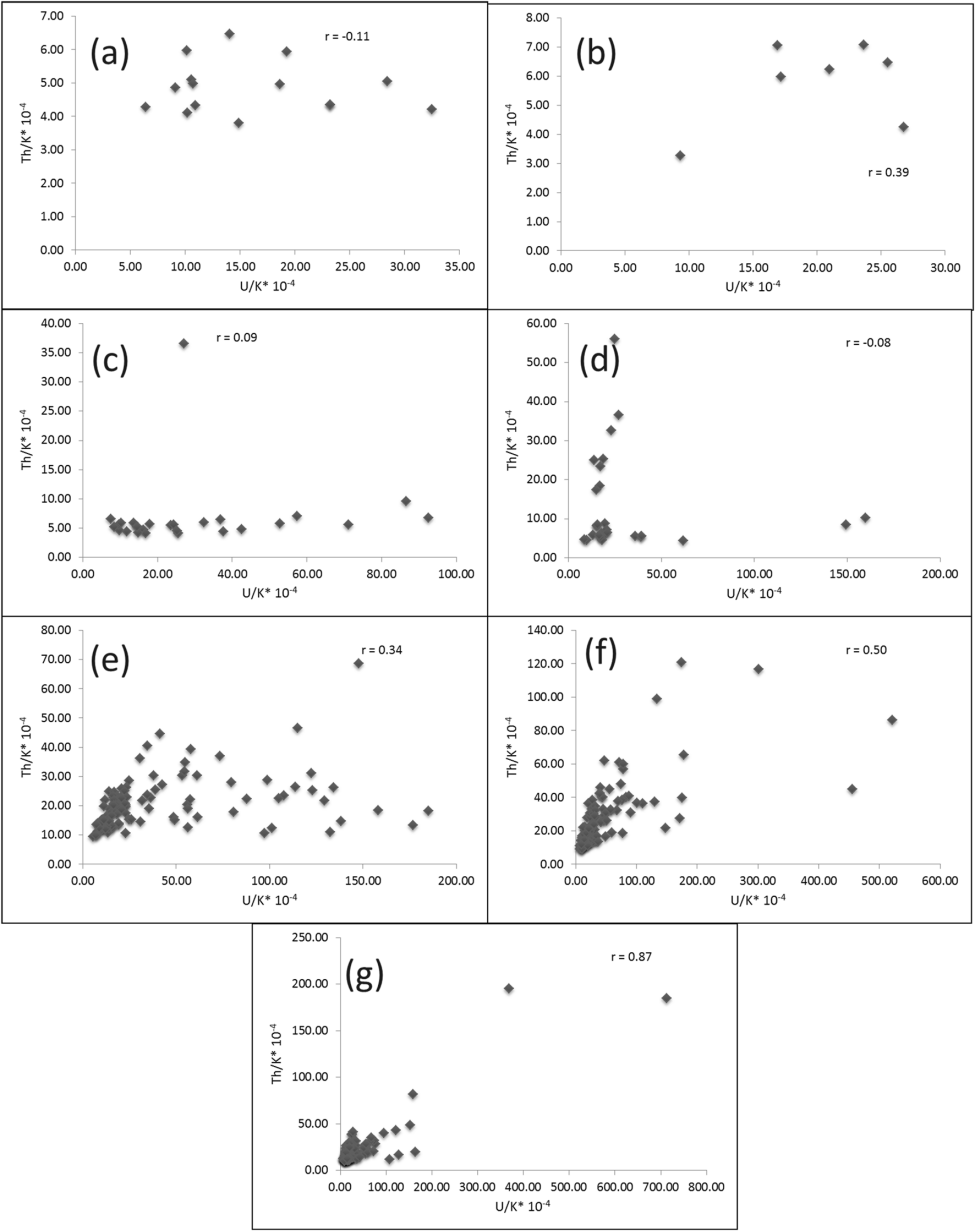
Binary plot of Th/K vs. U/K ratio showing weak correlations through hematitization (**a** and **b**-El-Erediya), red silica veins (**c** and **d**-El-Erediya), brecciated silica veins (**e** and **f**-El-Missikat), and massive silica veins (g-El-Missikat)

Th and K are concentrated during the late stages of magmatic differentiation, resulting in a strong positive correlation and 3–5*10^−4^Th/K ratios for the unaltered rocks [38, 64]. Through this context, Th values were plotted against K contents for the pink granite at El-Missikat and El-Erediya (Fig. 15a and b). This binary plot shows that the two elements are not related to each other (r = 0.14 and 0.15 for El-Missikat and El-Erediya, respectively), implying that the studied granite experienced either Th enrichment or K-metasomatism (potassic alteration). By using Th-K variation diagram constructed from the aforementioned normal ratios, the plotted samples of El-Missikat (Fig. 15c) and El-Erediya (Fig. 15d) pink granite are located through the normal range and the field of Th enrichment in which Th/K ratio > 5*10^–4^, while no samples are found through the field of K-metasomatism where Th/K<3*10^–4^. For shear zone, Th-enrichment is considered to be the dominant feature through El-Missikat alteration zones, including brecciation (Fig. 16a and b), greisenization (Fig. 16c and d), silicification (Fig. 16e), and kaolinization (Fig. 16f and g). On the other side, El-Erediya shear zone is characterized by the occurrence of Th enrichment along with normal Th/K ratio through the different alteration features, comprising brecciation (Fig. 17a and b), kaolinization (Fig. 17c and d), and hematitization (Fig. 17e and f). It is worth to mention that the occurrence of normal Th/K ratio through El-Erediya alteration zones likely indicates some pulses of the hydrothermal solutions caused K-metasomatism. Another genetic implication of Th/K ratio is revealed here by its relationship with U/K ratio. It was found that Th/K ratio has a strong positive correlation with U/K ratio through the studied alteration zones (Figs.18, 19) where thorite, zircon, betafite, and coffinite are common. On the other hand, hematitization, red and brecciated silica veins exhibit weak correlations (Fig. 20) and characterized by the abundance of secondary uranium minerals (e.g. uranophane and kasolite). K values are the same for the two ratios, so the strength of correlation indicate the extent to which U and Th mineralizations are associated with each other. In contrast to U, the weak mobilization of Th in supergene solutions causes much lower concentrations [60], and hence there is no chance to correlate with U. On the other side, hydrothermal U mineralization can be associated with hydrothermal Th mineralization (e.g. magmatic-hydrothermal solutions). At this point, it is claimed that Th/K vs. U/K can be used to discriminate between hydrothermal (strong positive correlation) and supergene uranium mineralizations (weak correlation).

## Conclusion

The detailed study of distribution of U, Th, and K as well as their ratios (Th/U, U/K, and Th/K ratios) through U mineralizations has proven its vital role in providing sufficient information to build-up a genetic scenario about magmatic, hydrothermal, and supergene U deposits. This was manifested by considering the shear zone-related U mineralizations through El-Missikat and El-Erediya exploratory tunnels, the Central Eastern Desert of Egypt, as a case study, where varied alteration zones, primary and secondary U mineralizations are well-exposed. Accordingly, our radiogenic ratios-based genetic scenario can be summarized through three main steps:

1. Tectonic shearing of the pink granite where many pathways become available for the upcoming mineralized solutions.
2. Many successive pulses of U and Th-bearing hydrothermal solutions, some of which are magmatic in origin, invaded the sheared granite and inferred by the weak correlation between U and Th (e.g. r = 0.5 and 0.04 for El-Missikat and El-Erediya granites, respectively) as well as Th and K (e.g. r = 0.14), 2.5 > Th/U > 0.1, Th/K > 5*10^–4^, the occurrence of thorite, columbite, xenotime and hydrothermal zircon (0.5 > Th/U ≤ 0.1), and the strong positive correlation between Th/K and U/K ratios (e.g. r = 0.74 and 0.80 in greisenized and kaolinized granite at El-Missikat and El-Erediya, respectively).
3. The hydrothermal U mineralization was then affected by the action of chemical weathering, resulting in the occurrence of secondary U minerals, including uranophane, kasolite, and zippeite, in places nearby the shear zone (e.g. brecciated and red silica veins and brecciated granite) where the supergene solutions readily percolate. This is reflected mainly by Th/U ratios ≤ 0.1 and the weak correlation between Th/K and U/K ratios (e.g. r = 0.34 and 0.08 for brecciated and red silica veins, respectively).

## Data Availability

All data are represented here.
